# Supplementary material for: A Critical View of the Application of the APEX Software (Aqueous Photochemistry of Environmentally-Occurring Xenobiotics) to Predict Photoreaction Kinetics in Surface Freshwaters
Source: Molecules. 2019 Dec 18;25(1):9. doi: 10.3390/molecules25010009 (PMC7017383; doi:10.3390/molecules25010009)
Supplement: Supplementary file 1 [file molecules-25-00009-s001.zip › 3.2.4_gcc-4.4.0.zip!/3.2.4_gcc-4.4.0/3.2.4_gcc-4.4.0/doc/octave/html/interpreter/Accessing-Global-Variables-in-Oct_002dFiles.html]

Accessing Global Variables in Oct-Files - Untitled


Next: Calling Octave Functions from Oct-Files,
Previous: Sparse Matrices in Oct-Files,
Up: Oct-Files


---

#### A.1.7 Accessing Global Variables in Oct-Files

Global variables allow variables in the global scope to be
accessed. Global variables can easily be accessed with oct-files using
the support functions `get_global_value` and
`set_global_value`. `get_global_value` takes two arguments,
the first is a string representing the variable name to obtain. The
second argument is a boolean argument specifying what to do in the case
that no global variable of the desired name is found. An example of the
use of these two functions is

```
```
     #include <octave/oct.h>
     
     DEFUN_DLD (globaldemo, args, , "Global demo.")
     {
       int nargin = args.length ();
       octave_value retval;
     
       if (nargin != 1)
         print_usage ();
       else
         {
           std::string s = args(0).string_value ();
           if (! error_state)
             {
               octave_value tmp = get_global_value (s, true);
               if (tmp.is_defined ())
                 retval = tmp;
               else
                 retval = "Global variable not found";
     
               set_global_value ("a", 42.0);
             }
         }
       return retval;
     }
```
```

An example of its use is

```
     global a b
     b = 10;
     globaldemo ("b")
     => 10
     globaldemo ("c")
     => "Global variable not found"
     num2str (a)
     => 42
```
